# Hypofractionated High-Dose Irradiation with Positron Emission Tomography Data for the Treatment of Glioblastoma Multiforme

**DOI:** 10.1155/2014/407026

**Published:** 2014-05-22

**Authors:** Kazuhiro Miwa, Masayuki Matsuo, Shin-ichi Ogawa, Jun Shinoda, Yoshitaka Asano, Takeshi Ito, Kazutoshi Yokoyama, Jitsuhiro Yamada, Hirohito Yano, Toru Iwama

**Affiliations:** ^1^Chubu Medical Center for Prolonged Traumatic Brain Dysfunction, Kizawa Memorial Hospital, Minokamo, Gifu 505-0034, Japan; ^2^Department of Neurosurgery, Chubu Medical Center for Prolonged Traumatic Brain Dysfunction, 630 Shimokobi, Kobi-cho, Minokamo, Gifu 505-0034, Japan; ^3^Department of Radiation Oncology, Kizawa Memorial Hospital, Minokamo, Gifu 505-0034, Japan; ^4^Department of Neurosurgery, Kizawa Memorial Hospital, Minokamo, Gifu 505-0034, Japan; ^5^Department of Neurosurgery, Gifu University Graduate School of Medicine, Gifu 501-1193, Japan

## Abstract

This research paper presents clinical outcomes of hypofractionated high-dose irradiation by intensity-modulated radiation therapy (Hypo-IMRT) with ^11^C-methionine positron emission tomography (MET-PET) data for the treatment of glioblastoma multiforme (GBM). A total of 45 patients with GBM were treated with Hypo-IMRT after surgery. Gross tumor volume (GTV) was defined as the area of enhanced lesion on MRI, including MET-PET avid region; clinical target volume (CTV) was the area with 5 mm margin surrounding the GTV; planning target volume (PTV) was the area with 15 mm margin surrounding the CTV, including MET-PET moderate region. Hypo-IMRT was performed in 8 fractions; planning the dose for GTV was escalated to 68 Gy and that for CTV was escalated to 56 Gy, while keeping the dose delivered to the PTV at 40 Gy. Concomitant and adjuvant TMZ chemotherapy was administered. At a median follow-up of 18.7 months, median overall survival (OS) was 20.0 months, and median progression-free survival was 13.0 months. The 1- and 2-year OS rates were 71.2% and 26.3%, respectively. Adjuvant TMZ chemotherapy was significantly predictive of OS on multivariate analysis. Late toxicity included 7 cases of Grade 3-4 radiation necrosis. Hypo-IMRT with MET-PET data appeared to result in favorable survival outcomes for patients with GBM.

## 1. Introduction

Glioblastoma multiforme (GBM) is the most common primary malignancy of the adult central nervous system (CNS) and is associated with an exceptionally poor prognosis. Although radiation therapy (RT) has been shown to prolong overall survival (OS) compared to surgery alone [1], patients treated with RT typically experience disease progression within the radiation field. GBMs are infiltrative tumors that usually spread through normal brain tissue, and it is difficult to demarcate glioma-affected areas from normal brain tissue.

Recently, new methods have been developed that enable elucidation of the biologic pathways of tumors, yielding additional information about the metabolism of the tumor tissue. Functional imaging studies, such as ^11^C-methionine positron emission tomography (MET-PET), have demonstrated increased metabolic activity due to increased amino acid transport in glioma cells compared to normal brain tissue [2]. Based on recent PET studies, we believe it would be reasonable to conduct a trial designed to evaluate the clinical outcome of RT selectively increasing the radiation dose to high-uptake area of MET-PET in patients with GBM.

Herein, we review our preliminary experience of planning and delivery of hypofractionated high-dose irradiation by intensity-modulated radiation therapy (Hypo-IMRT) with complementary use of MET-PET data. This study was designed to measure the acute and late toxicity of patients treated with our regimen, response of GBM to this treatment, OS, and the time to disease progression after treatment.

## 2. Material and Methods 

### 2.1. Patients

From April 2006 to July 2011, 45 patients with newly diagnosed GBM were enrolled. Eligibility criteria included histologically confirmed GBM, age ≥18 years, and adequate bone marrow, liver, and renal function. The extent of surgery was evaluated by three observers by viewing postoperative contrast-enhanced T1-weighted MRI images. Gross total resection of the tumor was defined as resection with no residual enhancing tumor. Exclusion criteria included multifocal or recurrent gliomas, involvement of the brainstem or posterior fossa, cerebrospinal fluid dissemination, severe concurrent disease, or prior history of RT or chemotherapy. Patients were grouped according to radiation therapy oncology group (RTOG) recursive partitioning analysis (RPA) class [3]. The institutional review board approved the study prior to patient enrollment. Informed consent was obtained from each subject after disclosing the potential risks of Hypo-IMRT and discussion of potential alternative treatments, including conventional three-dimensional conformal RT (3D-CRT). Patient characteristics are listed in Table 1.

#### 2.1.1. Imaging: CT

CT (matrix size: 512 × 512, FOV 50 × 50 cm) was performed using a helical CT instrument (Light Speed; General Electric, Waukesha, WI). Patient heads were immobilized in a commercially available stereotactic mask, and scans were performed with a 2.5 mm slice thickness without a gap.

#### 2.1.2. Imaging: MRI

MRI (matrix size: 256 × 256, FOV 25 × 25 cm) for radiation treatment planning was performed using a 1.5-T instrument (Light Speed; General Electric). Data were acquired using a standard head coil without rigid immobilization. An axial, three-dimensional gradient echo T1-weighted sequence with contrast medium and 2.0 mm slice thickness were acquired from the foramen magnum to the vertex, perpendicular to the main magnetic field.

#### 2.1.3. Imaging: MET-PET

The MET-PET study was performed using a standardized procedure. All patients fasted for at least 5 h before MET-PET and were advised to eat only a light breakfast in the morning of the examination day to ensure standardized metabolic conditions. The PET scanner was an ADVANCE NXi Imaging System (General Electric Yokokawa Medical System, Hino-shi, Tokyo), which provides 35 transaxial images at 4.25 mm intervals. The crystal width is 4.0 mm (transaxial). The in-plane spatial resolution (full width at half-maximum) was 4.8 mm, and scans were performed in standard two-dimensional mode. Before emission scans were performed, a 3-minute transmission scan was performed to correct photon attenuation using a ring source containing 68 Ge. A dose of 7.0 MBq/kg of MET was injected intravenously, depending on the exam. Emission scans were acquired for 30 min, beginning 5 min after MET injection. During MET-PET data acquisition, head motion was continuously monitored using laser beams projected onto ink markers drawn over the forehead skin and corrected as necessary. Image registrations were performed with Syntegra software (Philips Medical System, Fitchburg, WI) using a combination of automatic and manual methods. Automatic registration was performed, and three observers evaluated by consensus the fusion accuracy using landmarks such as the eyeball, lacrimal glands, and lateral ventricles.

### 2.2. Treatment

**Figure 1:**
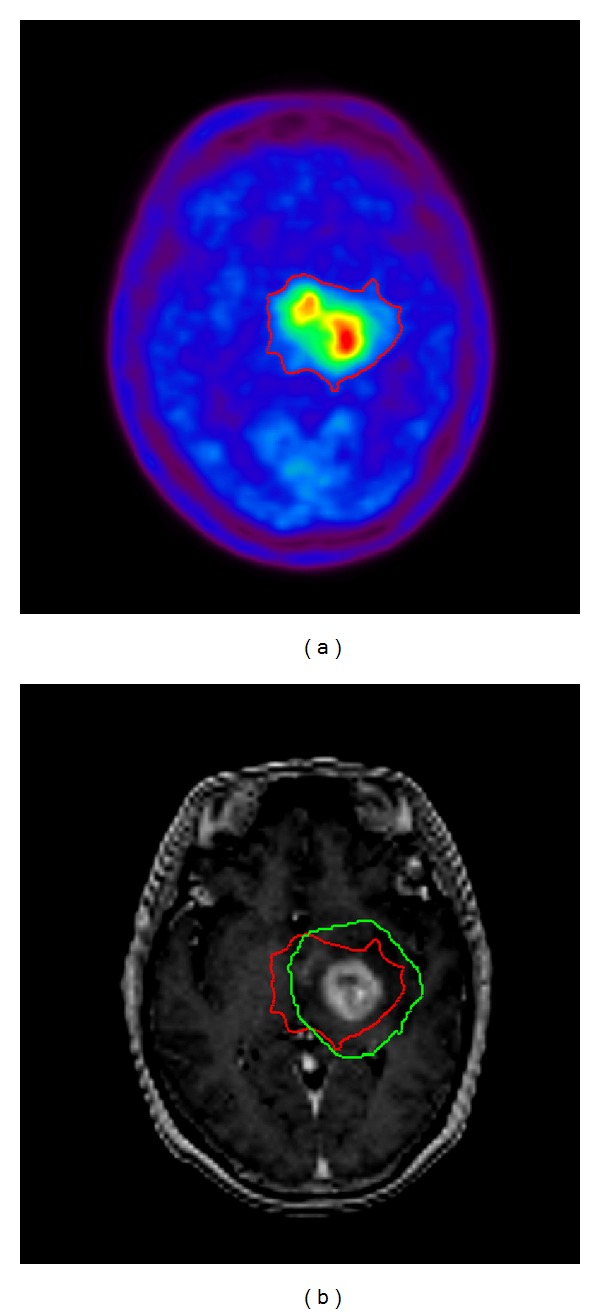
An example of the targets planned for a Hypo-IMRT procedure. (a) ^11^C-Methionine positron emission tomography (MET-PET). (b) Contrast-enhanced T1-weighted magnetic resonance imaging (MRI). Gross tumor volume (GTV) was the area of enhanced lesion on MRI, including MET-PET avid region. Clinical target volume (CTV) was the area with 5 mm margin surrounding the GTV. Both MET-PET moderate region (red line) and the area with 15 mm margin surrounding the CTV (green line) were included in planning target volume. The final determination of target delineation was obtained by consensus among three observers.

**Figure 2:**
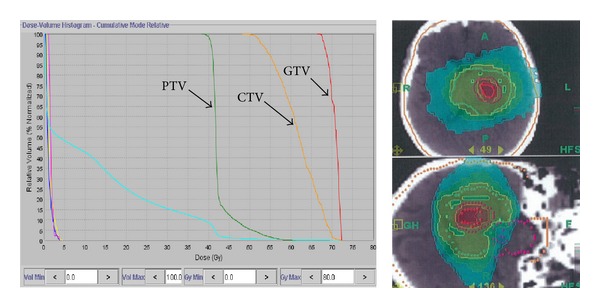
Dose map and dose-volume histogram of a representative case. Prescribed doses for gross tumor volume (GTV), clinical target volume (CTV), and planning target volume (PTV) were demonstrated.

Postoperative MRI and MET-PET were used along with the treatment-planning CT to define the radiation treatment volume. First, a simulation PET/MRI image fusion was performed for contouring. Secondly, the fusion image was positioned properly by CT scans equipped with tomotherapy (Helical TomoTherapy Hi-Art System, TomoTherapy Inc., Madison, WI). Three layered target volumes were contoured. Gross tumor volume (GTV) was the area of enhanced lesion on MRI, including MET-PET avid region; clinical target volume (CTV) was the area with 5 mm margin surrounding the GTV; planning target volume (PTV) was the area with 15 mm margin surrounding the CTV, including MET-PET moderate region (Figure 1). MET-PET avid region was defined, using a threshold value for the lesion versus normal counts of radioisotope per pixel (L/N) index of 1.7 or over. MET-PET moderate region was defined, using a threshold value for the L/N index of 1.3 or over. For primary brain tumors, both 1.3 [4] and 1.7 [5] have been used to determine the threshold value. Although we used 1.3 and 1.7 as the threshold for tumor delineation in this study, the final determination of MET-PET uptake region was confirmed by consensus among three observers. Hypo-IMRT was delivered using tomotherapy in 8 fractions. The dose for GTV was escalated to 68 Gy and that for CTV was escalated to 56 Gy as frequently as possible, while keeping the dose delivered to the PTV at 40 Gy. The dose was prescribed using the 95% isodose line, which covered PTV. Critical structures, including the brainstem, optic chiasm, lens, optic nerves, and cerebral cortex, were outlined, and dose-volume histograms for each structure were obtained to ensure that doses delivered to these structures were tolerable. The dose maps and dose-volume histograms of representative case are illustrated in Figure 2.

### 2.3. Chemotherapy

Patients received concomitant TMZ at a dose of 75 mg/m^2^ per day during Hypo-IMRT, followed by adjuvant TMZ at a dose of 150–200 mg/m^2^ per day for 5 days every 28 days, according to the European Organization for Research and Treatment of Cancer-National Cancer Institute of Canada regimen [6], starting 1 month after completion of RT. In the case of progression, patients were considered for second-line treatment on a case-by-case basis.

### 2.4. Follow-Up

Patients were assessed weekly during RT by clinical examination, complete blood count, blood chemistry, and liver enzyme tests. Regular follow-up was performed with serial neurological and radiological examinations at 1 month after completion of treatment and then every 3 months thereafter. Follow-up MRI and MET-PET were routinely conducted every 3 months or in the event of unexpected neurological worsening. When enlargement of the local lesion was observed, corticosteroid therapy was initiated and MRI was performed once a month thereafter to evaluate the efficacy of corticosteroid treatment. If the follow-up MRI revealed further enlargement of the enhanced mass, the lesion was diagnosed as “local progression,” and the day on which MRI first revealed lesion enlargement was defined as the date of progression. However, in cases in which a second surgery revealed no viable tumor cells in the enhanced lesion, the diagnosis was changed to “radiation necrosis.” Lesions that decreased in size during corticosteroid treatment were also defined as “radiation necrosis.” “Distant failure” was defined as the appearance of a new intraparenchymal enhanced lesion distant from the original tumor site. If the new lesion arose distant from the original tumor site and was exposed to the cerebrospinal fluid (CSF) space, the lesion was defined as “CSF dissemination.” Acute and late toxicities were scored according to RTOG criteria.

### 2.5. Statistical Analysis

Survival events were defined as death from any cause for OS and as disease progression for progression-free survival (PFS). OS and PFS were analyzed from the date of pathologic diagnosis to the date of the documented event using the Kaplan-Meier method. Tumor- and therapy-related variables were tested for a possible correlation with survival, using the log-rank test. Variables included Karnofsky performance status (KPS) (≥70 versus <70), RPA class (III, IV versus V, VI), surgery extent (gross total removal versus others), and adjuvant TMZ chemotherapy (yes versus no). The survival benefit was also evaluated by multivariate analysis using Cox's proportional hazards model.

## 3. Results

Patient characteristics are listed in Table 1. The median patient age was 61 years (range, 21–86 years). The average GTV was 12.11 ± 18.20 cm^3^, the average CTV was 43.64 ± 35.65 cm^3^, and the average PTV was 163.22 ± 100.15 cm^3^. The average GTV dose was 60.38 ± 5.25 Gy, and the average CTV dose was 53.15 ± 5.45 Gy. All 45 patients completed the prescribed Hypo-IMRT with concomitant TMZ course. Overall, 12 patients (26.7%) did not receive the prescribed course of adjuvant TMZ chemotherapy for the following reasons: neutropenia (*n* = 3), patient refusal (*n* = 4), nausea/vomiting (*n* = 2), and admission to another hospital (*n* = 3).

### 3.1. Toxicity Assessment

*Hematologic Toxicities*. Hematologic toxicities are listed in Table 2. During the acute phase, Grade 1-2 neutropenia occurred in 4 patients (8.9%). During the late phase, Grade 2-3 neutropenia occurred in 4 patients (8.9%), and Grade 4 thrombocytopenia occurred in 1 patient (2.2%).

*Nonhematologic Toxicities*. Nonhematologic toxicities are listed in Table 3. During the acute phase, nonhematologic toxicity was minimal, and no Grade 3-4 toxicities were reported. The most common acute toxicity reported was Grade 1-2 nausea/vomiting. Late nonhematologic toxicity included 7 cases of Grade 3-4 radiation necrosis (15.6%), 1 case of Grade 3 cerebropathy (2.2%), and 2 cases of Grade 3-4 intratumoral hemorrhage (4.4%). Median time to development of symptomatic radiation necrosis from the end of Hypo-IMRT was 9.3 months (range, 3–17 months). In seven cases of Grade 3-4 radiation necrosis, the average PTV was 152.04 ± 96.14 cm^3^, and the locations were one case of frontal lobe, three cases of temporal lobe, and three cases of parietal lobe. Grade 4 massive radiation necrosis required a second surgery in 2 cases (4.4%) 8 and 14 months after Hypo-IMRT. These patients are alive without disease for 13 and 11 months, respectively, after necrotomy. In 5 cases with Grade 3 radiation necrosis, initiation or increased dosage of corticosteroid therapy was required for worsening of neurological symptoms. One of these five patients died of disseminated disease 23 months after Hypo-IMRT, and the remaining four patients are alive without disease for 14, 17, 18, and 39 months after Hypo-IMRT. Although the correlation with treatment is unclear, Grade 3-4 intratumoral hemorrhage was observed 13 and 21 months after IMRT in 2 cases. The representative case of radiation necrosis was demonstrated in Figure 5.

### 3.2. Outcomes

**Figure 3:**
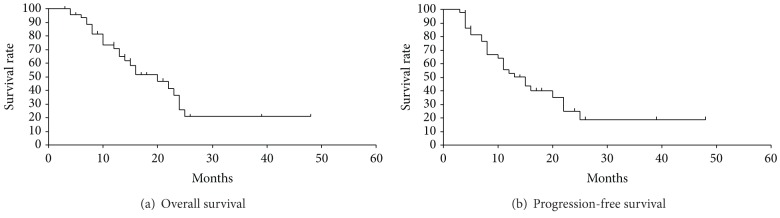
Overall survival (a) and progression-free survival (b) for all patients. Median OS was 20.0 months, and the 1- and 2-year OS rates were 71.2% and 26.3%, respectively. Median PFS was 13.0 months, and the 1- and 2-year PFS rates were 52.6% and 20.6%, respectively.

**Figure 4:**
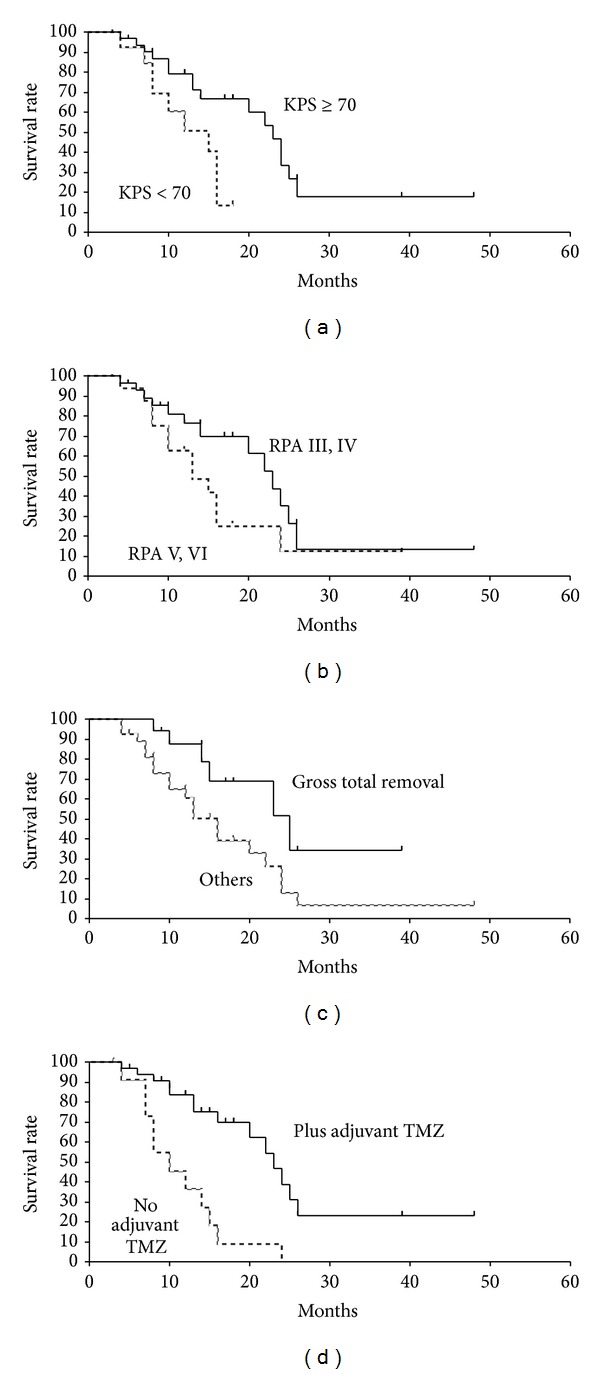
Overall survival rates among different subgroups by (a) Karnofsky performance status (KPS), (b) recursive partitioning analysis (RPA) subclass, (c) extent of surgical resection, and (d) adjuvant temozolomide (TMZ) chemotherapy.

*Overall Survival*. At a median follow-up of 18.7 months (range, 3–48 months), median OS was 20.0 months (range, 3–48 months). The 1- and 2-year OS rates were 71.2% and 26.3%, respectively (Figure 3(a)). The survival rates by KPS, RPA class, extent of surgical resection, and adjuvant TMZ chemotherapy are shown in Figures 4(a)–4(d). KPS (≥70 versus <70), extent of surgical resection (gross total removal versus others), and adjuvant TMZ chemotherapy (yes versus no) were significant predictive factors of OS as tested by univariate analysis. Multivariate analysis revealed only adjuvant TMZ chemotherapy to be a significant variable predictive of OS (Table 4).

*Progression-Free Survival*. Median PFS was 13.0 months (range, 3–48 months), and the 1- and 2-year PFS rates were 52.6% and 20.6%, respectively (Figure 3(b)). CSF dissemination was the most frequent failure pattern, which was observed in 17 cases (60.7% of all failures). Local progression was observed in 7 patients (25.0%), and distant failure was observed in 4 cases (14.3%). An example of a failure with CSF dissemination after Hypo-IMRT is shown in Figure 6. KPS (≥70 versus <70) was the only predictive factor for PFS as tested by univariate analysis. On multivariate analysis, none of the variables were significantly predictive of PFS (Table 5).

## 4. Discussion

Lately, much work has been performed on various hypofractionation regimens and dose escalation with Hypo-IMRT for GBM which revealed relatively favorable survival results [7–11], although a distinct advantage over conventional RT has not been demonstrated. However, if a very conformal treatment technique such as Hypo-IMRT can be implemented and a greater biologic dose to the infiltrating tumor is possible through hypofractionation, it could be possible to deliver a more effective therapy that may increase patient survival without increasing morbidity. To meet these requirements, the contouring of the target volume must be of critical importance in the treatment of Hypo-IMRT.

PET is a newer method that can improve the visualization of molecular processes. In one study, several amino acids were radio-labeled to evaluate their potential imaging characteristics in primary brain tumors; such an analysis might be expected to elucidate mechanisms related to either amino acid metabolism or breakdown of the BBB [2]. In recent PET studies, analysis of the metabolic and histologic characteristics of stereotactic biopsy specimens provided evidence that regional high MET uptake correlates with the malignant pathologic features [4, 12, 13]. In many cases of malignant glioma, the size and location of MET uptake differ considerably from the abnormalities observed on CT/MRI [14–16]. Matsuo et al. reported that MET-PET had promising potential for precisely delineating target volumes in planning radiation therapy for postoperative patients with GBM [16]. Lee et al. reported the results of a study demonstrating statistically significant correlation between the presence of increased MET-PETehl uptake outside the high-dose region and subsequent noncentral failure [17].

Considering the informative results of these recent trials, MET-PET might have substantial reliability as a marker of tumor biological characteristics, as well as a valuable impact on visualizing the tumor-invasive area of malignant glioma. Previously, we reported three preliminary cases of GBM treated by Hypo-IMRT with complementary use of MET-PET [18]. Herein, we report the results of prospective study to evaluate the clinical outcome of Hypo-IMRT selectively increasing the radiation dose to MET-PET uptake region (Figure 1). Consequently, we found the treatment outcomes demonstrating that median OS was 20.0 months (range, 3–48 months) and that the 1- and 2-year OS rates were 71.2% and 26.3%, respectively (Figure 3(a)). Median PFS was 13.0 months (range, 3–48 months), and the 1- and 2-year PFS rates were 52.6% and 20.6%, respectively (Figure 3(b)). Survival results of our study appeared to be favorable to published results using standard fractionation RT combined with TMZ [6]. Needless to say, direct comparisons to historical controls or to other studies should be viewed with caution; only a properly designed randomized trial can firmly establish whether the present regimen is superior to the standard treatment.

In the special feature of this study, local tumor progression occurred at a lower incidence (25.0%), suggesting that selectively increasing the radiation dose to MET-PET uptake area contributed to better local control of original tumors. Meanwhile, the most common type of failure was CSF dissemination (60.7% of all failures). It still remains difficult to prevent CSF dissemination for extended periods with our regimen, although the original lesion might be well controlled (Figure 6). For improved patient survival, prevention of CSF dissemination may be the next issue to be addressed. If new, targeted chemotherapeutic agents lead to further improvements in control of microscopic disease, radiation can be used primarily to control disease in limited regions that have the highest risk of progression, that is, where those agents are most likely to fail.

Both univariate analysis and multivariate analysis revealed a significant difference in OS between patients who did and did not receive adjuvant TMZ (Table 4). We estimated that the addition of TMZ might be particularly effective if the radiation dose to normal brain tissue was limited by better targeting. However, the impact of TMZ along with the methylation status of the O-6-methylguanine-DNA methyltransferase (MGMT) on survival was not systematically evaluated in the present study. This selection bias could not be avoided, as the patients with methylated MGMT or who were in better clinical condition were more likely to have received adjuvant TMZ chemotherapy. Nevertheless, the results of this study support our initial work and further establish the efficacy of this regimen combined with TMZ.

**Figure 5:**
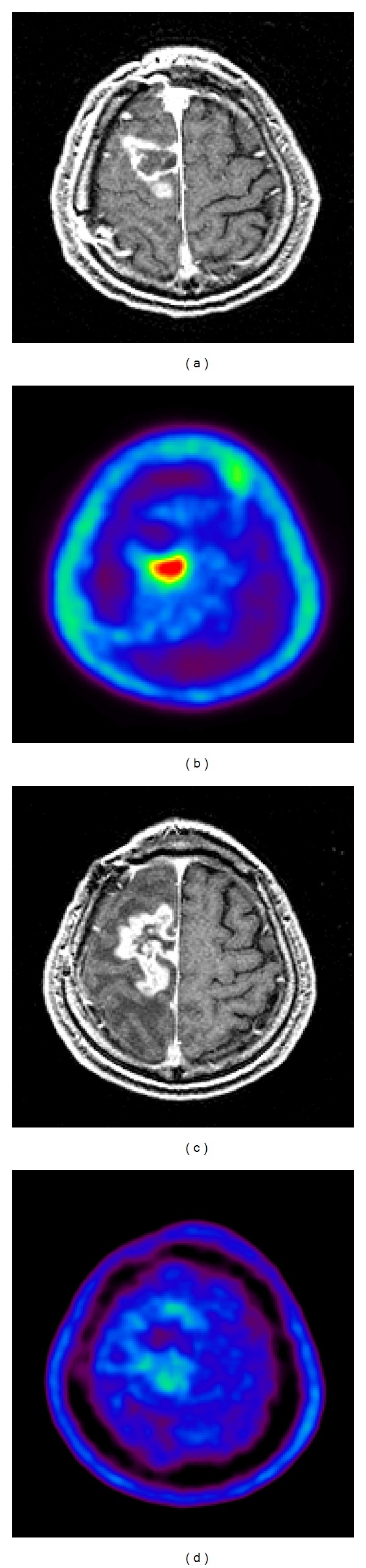
A 56-year-old man of GBM with symptomatic radiation necrosis. Before Hypo-IMRT, enhanced lesions were demonstrated in the right frontal lobe on T1-weighted magnetic resonance imaging (MRI) (a). ^11^C-Methionine positron emission tomography (MET-PET) demonstrated a MET high-uptake on the region (b). 12 months after Hypo-IMRT, the enhanced lesion with perifocal edema was increased in size (c), although MET uptake decreased in the irradiated region (d). Second surgery was performed, and pathological diagnosis was defined as necrotic tissue without viable tumor cells.

**Figure 6:**
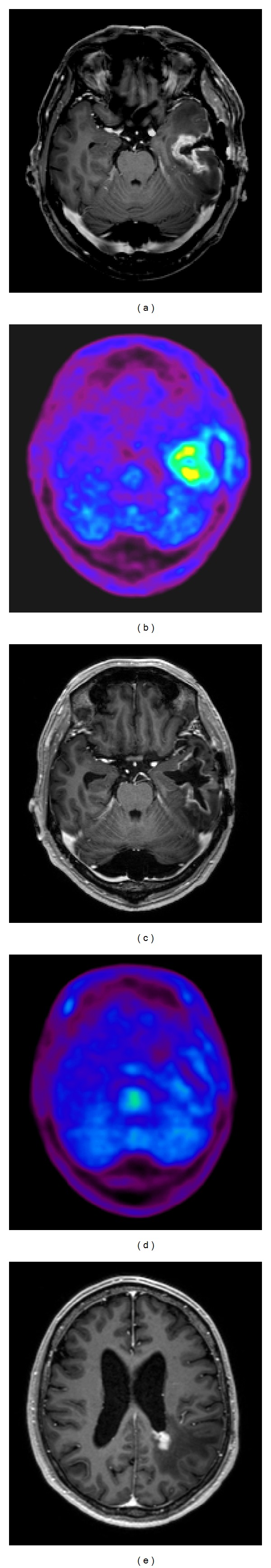
Representative cases of cerebrospinal fluid dissemination: 55-year-old man with GBM. Before Hypo-IMRT, an enhanced tumor was demonstrated in the left temporal lobe on T1-weighted magnetic resonance imaging (MRI) (a). ^11^C-Methionine positron emission tomography (MET-PET) demonstrated a MET high-uptake region in the left temporal lobe (b). 20 months after Hypo-IMRT, the enhanced tumor was decreased in size (c), and the MET high-uptake region could not be detected distinctly (d), although disseminated lesion was observed around the left lateral ventricle (e).

Late toxicity was more common with this treatment regimen than early toxicity; specifically, the most severe adverse event associated with our regimen was radiation necrosis. Overall, 5 and 2 patients experienced Grade 3 and Grade 4 radiation necrosis, respectively, and necrotomy was required in 2 patients (Figure 5). These 2 patients are alive without disease for 11 and 13 months after necrotomy. In another 5 cases with Grade 3 radiation necrosis, 4 patients are alive without disease for 14, 17, 18, and 39 months after Hypo-IMRT. Recently, the efficacy of new chemotherapeutic agents, such as bevacizumab, which are used as rescue therapies for radiation necrosis after RT, has been reported [19, 20]. It is possible that if a rescue therapy to prevent radiation necrosis was conducted on a larger scale with a sufficient number of patients, a more accurate conclusion about patient outcome could be made.

## 5. Conclusion

Our preliminary study demonstrated that Hypo-IMRT with complementary use of MET-PET data appeared to result in favorable survival outcomes for patients with GBM, although a properly designed randomized trial could firmly establish whether the present regimen is superior to the standard treatment.

## Figures and Tables

**Table 1:** Patient's characteristics.

Parameter	*n* (%)
Age, years	
≥50	37 (82.2)
<50	8 (17.8)
Gender	
Male	28 (62.2)
Female	17 (37.8)
KPS score	
≥70	31 (68.9)
<70	14 (31.1)
RPA class	
III	5 (11.1)
IV	23 (51.1)
V	5 (11.1)
VI	12 (26.7)
Resection	
GTR	17 (37.8)
Others	28 (62.2)
Adjuvant TMZ chemotherapy	
Yes	33 (73.3)
No	12 (26.7)

KPS: Karnofsky performance status, RPA: recursive partitioning analysis, GTR: gross total removal, and TMZ: temozolomide.

**Table 2:** Hematologic toxicity.

Adverse event	Acute phase				Late phase			
	G 1	G 2	G 3	G 4	G 1	G 2	G 3	G 4
Neutropenia	1 (2.2)	3 (6.7)	0 (0)	0 (0)	0 (0)	3 (6.7)	1 (2.2)	0 (0)
Thrombocytopenia	0 (0)	0 (0)	0 (0)	0 (0)	0 (0)	0 (0)	0 (0)	1 (2.2)

G: grade.

Data presented as number of patients, with percentages in parentheses.

**Table 3:** Nonhematologic toxicity.

Adverse event	Acute phase				Late phase			
	G 1	G 2	G 3	G 4	G 1	G 2	G 3	G 4
Headache	3 (6.7)	0 (0)	0 (0)	0 (0)	0 (0)	0 (0)	0 (0)	0 (0)
Nausea/vomiting	2 (4.4)	2 (4.4)	0 (0)	0 (0)	0 (0)	0 (0)	0 (0)	0 (0)
Radiation necrosis	0 (0)	0 (0)	0 (0)	0 (0)	0 (0)	0 (0)	5 (11.1)	2 (4.4)
Cerebropathy	0 (0)	0 (0)	0 (0)	0 (0)	0 (0)	0 (0)	1 (2.2)	0 (0)
Hemorrhage	0 (0)	0 (0)	0 (0)	0 (0)	0 (0)	0 (0)	1 (2.2)	1 (2.2)

G: grade.

Data presented as number of patients, with percentages in parentheses.

**Table 4:** Analysis of prognostic variables for overall survival.

Variables	Median survival (months)	Univariate analysis* *P* value	Multivariate analysis^†^ *P* value
KPS			
≥70	23	0.0297	0.5994
<70	15		
RPA-class			
III, IV	23	0.1385	
V, VI	13		
Extent of resection			
GTR	25	0.0422	0.131
Others	13		
Adjuvant TMZ chemotherapy			
Yes	23	0.0004	0.0124
No	10		

Abbreviations as in Table 1.

Statistical analyses were performed with*log-rank test and ^†^Cox's proportional hazards model.

**Table 5:** Analysis of prognostic variables for progression-free survival.

Variables	Median survival (months)	Univariate analysis* *P* value	Multivariate analysis^†^ *P* value
KPS			
≥70	20	0.0282	0.1279
<70	8		
RPA-class			
III, IV	16	0.5895	
V, VI	11		
Extent of resection			
GTR	22	0.2004	
Others	11		
Adjuvant TMZ chemotherapy			
Yes	16	0.0548	
No	8		

Abbreviations as in Table 1.

Statistical analyses were performed with *log-rank test and ^†^Cox's proportional hazards model.
